# Endovascular Approach to Severe Acute Type B Aortic Dissection

**DOI:** 10.7759/cureus.6528

**Published:** 2019-12-31

**Authors:** João Vítor Ternes Rech, Caique Martins Pereira de Moura Ternes, Gustavo Busch Justino, Rafael Narciso Franklin, Gilberto Do Nascimento Galego

**Affiliations:** 1 Surgery, Federal University of Santa Catarina, Florianópolis, BRA; 2 Medicine, Federal University of Santa Catarina, Florianópolis, BRA; 3 Cardiac/Thoracic/Vascular Surgery, Federal University of Santa Catarina, Florianópolis, BRA

**Keywords:** aortic dissection, stanford type b, endovascular, debakey iii

## Abstract

Acute aortic dissection (AAD) is an important emergency that should be identified promptly. The classification of AAD follows two different systems: Stanford (which defines lesions as types A, on the ascending aorta, or B, on the descending aorta) and DeBakey, which also accounts for the extension of the aortic dissection. We present a notable case of a 63-year-old male who presented with a history of abrupt abdominal pain radiating to the dorsal region for endovascular treatment. He was oliguric with symmetric pulses in the superior limbs and reduction of pulses in the left lower limbs, with signs of hypoperfusion. Angiotomography evidenced acute abdominal thoracic aortic dissection classified as DeBakey III and Stanford B, extending through the left iliac artery. He was submitted to endovascular correction of the abdominal thoracic aortic dissection, with implantation of two straight Valiant type endoprosthesis (26x200 mm and 38x200 mm), positioned after the emergence of the left subclavian artery and right above the celiac trunk, respectively. There was also implantation of the stent graft Viabahn (5x60 mm) and Assurant stent (7x30 mm) in the left renal artery. After the urgent surgical intervention, the patient has recovered well. He has been checked in outpatient follow-ups for the past three years with preserved renal function (1.5 mg/dl creatinine) and correct positioning of the endoprosthesis (confirmed by CT without contrast). Hypertension and a smoking history are the most important risk factors associated with aortic dissections, and should be considered when evaluating a patient with chest or back pain (typically described as sharp rather than tearing or ripping) in the emergency department. The endovascular approach to descending dissections was introduced in 1999 and has been established as the standard approach to descending dissections of the aorta, because of the excess mortality of the open approach (32% in open surgery and 7% for those managed with endovascular techniques) and low rate of complications. Ten-year survival rates for patients with AAD ranging from 30% to 60% justifies an aggressive follow-up strategy of discharge, with the goal of minimizing aortic wall stress through drugs (such as β blockers) and surveillance to detect progression. Our report shows that an early detection of symptoms coupled with an aggressive and precise endovascular intervention has produced satisfactory clinical, laboratorial and quality-of-life outcomes in an older patient with an extensive type B arterial dissection.

## Introduction

Acute aortic dissection (AAD) is a life-threatening emergency defined as an intimal tearing of the aortic wall, which allows for the flow of blood from the true lumen to a newly formed false lumen located between the intimal and medial layers of the aorta [1]. The high pressure within the aorta may then cause continuous flow through this entry tear, causing propagation of the false lumen anterograde or retrograde along the aorta (or both), finally generating disruption of blood flow and progressive weakness of the aortic wall, which could lead to aortic rupture [2].

The classification of AAD follows two different systems: Stanford (which defines lesions as types A, on the ascending aorta, or B, on the descending aorta) and DeBakey, which also accounts for the extension of the aortic dissection. Clinical symptoms include chest or back pain, typically described as sharp (rather than tearing or ripping), are the most common presenting complaints in the emergency room, which should be identified promptly in order to prevent mortality [1,2]. We herein present a case of an older male with extensive type B aortic dissection referred to Coris Medical Clinic, located in Florianópolis, Brazil.

## Case presentation

**Figure 1:**
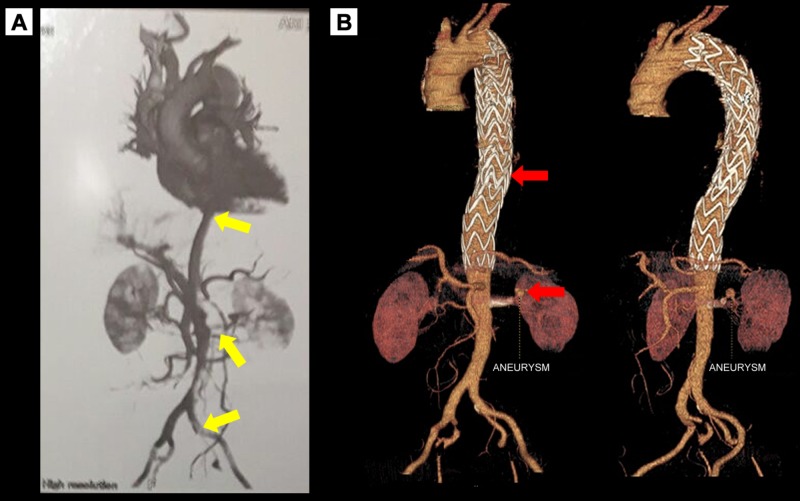
Preoperative CTA acute showing aortic dissection type B with exclusion of the left kidney, reduced lumen of the infrarenal aorta and iliac arteries, with discreet contrast filling of the left iliac artery indicated by yellow arrows (A) and three-dimensional CTA reconstruction showing postoperative endoprosthesis placement and a left renal artery aneurysm marked by the red arrows (B). CTA: computed tomography angiography

A 63-year-old male with a history of hypertension and tabagism presents with a complaint of abrupt abdominal pain radiating to the dorsal region for endovascular treatment. Although hemodynamically stable on admission, he was oliguric, with symmetric pulses in the superior limbs and reduction of pulses in the left lower limbs, with signs of hypoperfusion, in the physical exam. Angiotomography evidenced acute abdominal thoracic aortic dissection classified as DeBakey III and Stanford B, extending through the left iliac artery (Figure 1A). He was submitted to endovascular correction of the abdominal thoracic aortic dissection, with implantation of two straight Valiant type endoprosthesis (26x200 mm and 38x200 mm), positioned after the emergence of the left subclavian artery and right above the celiac trunk, respectively. There was also implantation of the stent graft Viabahn (5x60 mm) and Assurant stent (7x30 mm) in the left renal artery (Figure 1B).

**Figure 2:**
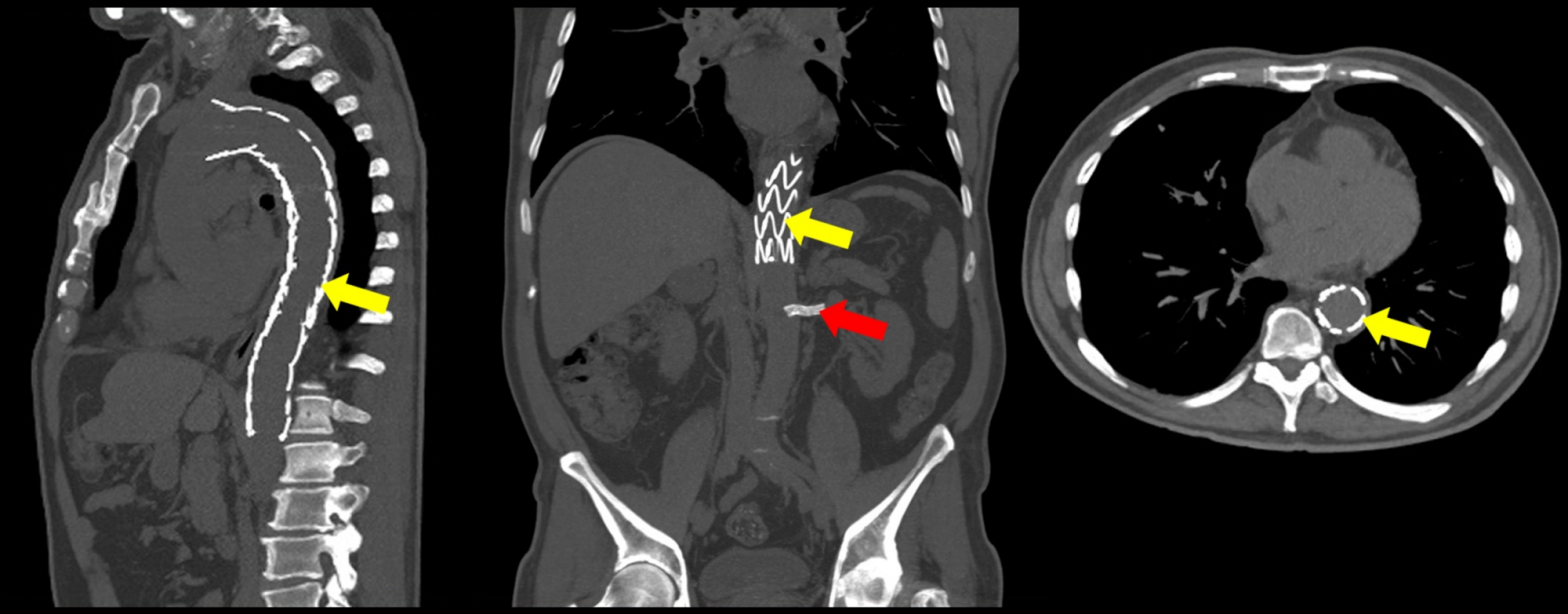
Follow-up sagittal, coronal and axial planes CT showing correct placement of the two Valiant type endoprosthesis after the emergence of the left subclavian artery and right above the celiac trunk, respectively (yellow arrows), and the Viabahn and Assurant stents in the left renal artery (red arrow). CT: computed tomography

The patient presented well in the postoperative period, with successful recovery of the renal function. He stayed in the ICU for the treatment of resistant hypertension and of an infection and then was discharged from the ICU on the 11th postoperative day and from the hospital in the 18th day, with adequate pressoric control and 2 mg/dl creatinine. He has been checked in outpatient follow-ups for the past three years with preserved renal function (1.5 mg/dl creatinine) and correct positioning of the endoprosthesis, followed by CT without contrast (Figure 2).

## Discussion

Different studies suggest an incidence of acute aortic dissection ranged from 2.9 to 3.5 per 100,000 per year, while the average age of patients afflicted ranged from 48 to 67 years (median age, 61 years), with 50% to 81% of them being male. Hypertension and a smoking history were the most important risk factors associated with aortic dissections, followed by hyperlipidemia, atherosclerosis, cocaine or stimulant use, deceleration trauma and a variety of conditions that decrease the strength of the aortic wall (such as Marfan syndrome and Ehler-Danlos syndrome), as well as vasculitides (such as Takayasu arteritis and Behçet’s disease) [1,3].

The endovascular approach to descending dissections was introduced in 1999 and has been established as the standard approach to descending dissections of the aorta, because of the excess mortality of the open approach (32% in open surgery and 7% for those managed with endovascular techniques) and low rate of complications. Ten-year survival rates for patients with AAD ranging from 30% to 60% justifies an aggressive follow-up strategy of discharge, with the goal of minimizing aortic wall stress through drugs (such as β blockers) and surveillance to detect progression [3,4]. Open repair, although still practiced in many parts of the world, is currently indicated only for those patients with contraindications or infeasibility of endovascular intervention, and it involves the replacement of the descending aorta with a graft, with excision of the intimal tear and restoration of perfusion in order to prevent aortic rupture [5].

## Conclusions

Type B aortic dissection is an important and life-threatening condition that requires a specific treatment modality depending on disease presentation. High-risk patients (such as those with hypertension and a smoking history) presenting with a sharp chest or back pain are to be attentively evaluated. Our case stresses that an early detection of symptoms coupled with an aggressive and precise endovascular intervention is crucial to produce satisfactory clinical, laboratorial and quality-of-life outcomes in an older patient with an extensive type B arterial dissection, improving survival outcomes.
